# P10 *Klebsiella* CP-CRE infection outbreak among liver and kidney transplant recipients—a single centre experience

**DOI:** 10.1093/jacamr/dlaf230.017

**Published:** 2025-12-04

**Authors:** Alif Adlan Mohd Thabit, Anuradha P Radhakrishnan, Farina Mohamad, Norazlah Bahari, Ahneez Abdul Hameed

**Affiliations:** Infectious Diseases Unit, Selayang Hospital, Batu Caves, Malaysia; Infectious Diseases Unit, Selayang Hospital, Batu Caves, Malaysia; Infection Control Unit, Selayang Hospital, Batu Caves, Malaysia; Microbiology Unit, Selayang Hospital, Batu Caves, Malaysia; Microbiology Unit, Selayang Hospital, Batu Caves, Malaysia

## Abstract

**Background:**

In Malaysia, the most common carbapenem-resistant Enterobacterales (CRE) species is *Klebsiella* spp. (63.8%), and the New Delhi MBL 1 (NDM-1) gene was most prevalent (83.6%). We report 11 carbapenemase-producing carbapenem-resistant Enterobacterales (CP-CRE) *Klebsiella* cases among 11 patients—8 kidney and 3 liver transplant patients—in Selayang Hospital from September to December 2023.

**Objectives:**

To control the CP-CRE outbreak and to prevent further transmission of CP-CRE cases.

**Methods:**

An urgent ad hoc meeting with the Hospital Infection and Antibiotic Control Committee (HIACC) and heads of departments of concern was organized with the hospital infection and prevention control unit (IPC). Daily audits by the infectious diseases team followed by Continuous Medical Education (CME) and Continuous Nursing Education (CNE) with the respective departments, PFGE use to identify transmission links for targeted intervention, and patient awareness through a pilot educational video and printed handouts were implemented.

**Results:**

All the CP-CRE Klebsiella cases were tested NDM-1 strain via the PCR method. Selected positive CP-CRE isolates on culture during a 1 week period were sent for PFGE testing including 3 kidney and 1 liver transplant recipients. Two of the three kidney transplant recipients shared similar PFGE signatures—Patients 7 and 8 showed 85.7% genetic similarities of Pattern A between them. By the end of December 2023, CP-CRE *Klebsiella* infection cases from both renal and hepatobiliary departments had ceased after strict infection control interventions were enforced.

**Conclusions:**

We find that PFGE and patient awareness thru printed and visual education are useful tools for evaluating the presence of horizontal transmission in hospital-acquired infection and as part of outbreak control measures respectively.

